# Reduction of peritoneal carcinomatosis by intraperitoneal administration of phospholipids in rats

**DOI:** 10.1186/1471-2407-7-104

**Published:** 2007-06-21

**Authors:** Jens Otto, Petra Lynen Jansen, Stefan Lucas, Volker Schumpelick, Marc Jansen

**Affiliations:** 1 Department of Surgery, University Clinic RWTH Aachen, Pauwelsstrasse 30, 52057 Aachen, Germany

## Abstract

**Background:**

Intraperitoneal tumor cell attachment after resection of gastrointestinal cancer may lead to a developing of peritoneal carcinosis. Intraabdominal application of phospholipids shows a significant decrease of adhesion formation even in case of rising tumor cell concentration.

**Methods:**

In experiment A 2*10^6 ^colonic tumor cells (DHD/K12/Trb) were injected intraperitonely in female BD-IX-rats. A total of 30 rats were divided into three groups with treatments of phospholipids at 6% or 9% and the control group. In experiment B a total of 100 rats were divided into ten groups with treatments of phospholipids at 9% and the control group. A rising concentration of tumor cells (10,000, 50,000, 100,000, 250,000 and 500,000) were injected intraperitonely in female BD-IX-rats of the different groups. After 30 days, the extent of peritoneal carcinosis was determined by measuring the tumor volume, the area of attachment and the Peritoneal Cancer Index (PCI).

**Results:**

In experiment A, we found a significant reduction (control group: tumor volume: 12.0 ± 4.9 ml; area of tumor adhesion: 2434.4 ± 766 mm^2^; PCI 28.5 ± 10.0) of peritoneal dissemination according to all evaluation methods after treatment with phospholipids 6% (tumor volume: 5.2 ± 2.2 ml; area of tumor adhesion: 1106.8 ± 689 mm^2^; PCI 19.0 ± 5.0) and phospholipids 9% (tumor volume: 4.0 ± 3.5 ml; area of tumor adhesion: 362.7 ± 339 mm^2^; PCI 13.8 ± 5.1). In experiment B we found a significant reduction of tumor volume in all different groups of rising tumor cell concentration compared to the control. As detected by the area of attachment we found a significant reduction in the subgroups 1*10^4^, 25*10^4 ^and 50*10^4^. The reduction in the other subgroups shows no significance. The PCI could be reduced significantly in all subgroups apart from 5*10^4^.

**Conclusion:**

In this animal study intraperitoneal application of phospholipids resulted in reduction of the extent of peritoneal carcinomatosis after intraperitoneal administration of free tumor cells. This effect was exceptionally noticed when the amount of intraperitoneal tumor cells was limited. Consequently, intraperitoneal administration of phospholipids might be effective in reducing peritoneal carcinomatosis after surgery of gastrointestinal tumors in humans.

## Background

Peritoneal carcinosis can be a result of intraperitoneal tumor cell spread after surgical treatment of colonic cancer. Tumor cell attachment occurs through blood-or lymphatic vessels or by accidentally opening the colonic specimen [1]. Serosal invasion of the primary tumor leads in up to 50% of patients to intraperitoneal metastases [2]. However, even stage 1 and stage 2 of colonic cancer, gastric cancer, uterine cancer or pancreatic cancer may cause peritoneal dissemination [2]. In gastrointestinal cancer the detection of free intraperitoneal tumor cells, serves as an independent prognostic factor [3]. Free floating intraperitoneal tumor cells may attach to, degrade and migrate through the extracellular matrix (ECM) [4]. Particularly if the peritoneum is damaged and the etracellular matrix is exposed tumor cell adhesion accumulates [5]. In former studies we found a widespread peritoneal carcinosis, with tumor cell adhesion in most peritoneal aeras after intraabdominal instillation of tumor cells (Cell line: DHD/K12/TRb). We could demonstrate that tumor cells predominantly adhere to injured peritoneal areas. However, the goal of the underlying study was not locoregional recurrence but the effect of phospholipids in the complete peritoneal cavity. In addition preliminary studies could show that a phospholipid emulsion is able to significantly reduce intraperitoneal tumor cell adhesion [3,6,7].

Phospholipids are natural constituents of peritoneal fluid secreted by mesothelial cells. These polar phosphoric acid di-esters are capable to form a lubricant layer on the peritoneal surface, which is of paramount importance to prevent adhesion [19,20]. Additionally, treatment with phospholipids (e.g. gangliosides) affect integrin function, causing reduced cell motility and adhesion capability after exogenous addition of phospholipids [8-10]. Furthermore other adhesion-preventing substances are known. For example Jeekel et al. evaluated the effects of intra-abdominal treament with Icodextrin, a glucose polymer solution, in a coloncarcinoma CC531 rat model [11].

As we could demonstrate the positive effect of phospholipids in low dosage in former studies, the aim of the underlying experiment was to compare the influence of rising phospholipid concentrations on the one hand and different tumor cell concentrations on the other hand with special emphasis on possible side effects.

## Methods

### Animals and anesthesia

In this study a total of 130 female BD-IX rats (mean body weight 200 g +/- 10 g) were operated. The animals were kept under standard laboratory conditions with free access for food and water entire study, which was performed according to the rules of the "Deusche Tierschutzgesetz" (50.203.AC 18, 9/02) and to the guidelines for the use of laboratory animals. The animals were assigned to the following groups of 10 rats (Table 1). The surgical procedure was performed under sterile conditions and general anesthesia by intramuscular injection of ketamine (100 mg/kg Bodyweight BW) (Ketamin 10%, Sanofi-Cefa, Düsseldorf, Germany) and rompun 2% (5 mg/kg BW) (Rompun 2%, Bayer, Leverkusen, Germany).

**Table 1:** Arrangement of animal groups in experiment A

Experiment A	control	PL 6%	PL 9%	period
	10 rats	10 rats	10 rats	30 days

(PL = phospholipids)

### Tumor cell culture

Colonic adenocarcinoma induced in syngenic female BD-IX rats is the source of the cell line (DHD/K12/TRb) used in this investigation [5]. Cells were obtained from the European Collection of Animal Cell Cultures (ecacc, Salisbury, UK). They were cultivated in monolayers in tissue culture flasks (75 cm^2^, Falcon, Becton Dickinson, Heidelberg, Germany) in DMEM and Ham's F10 (1:1; GIBCO) supplemented with 10% fetal bovine serum (GIBCO) and gentamycin (0,005%; GIBCO). Cells were incubated at 37°C in a humidified atmosphere of 5% CO_2_. They were passaged after treatment with 0,125% trypsin for 2 min. Following centrifugation for 10 min at 200 g, cells were suspended in 20 ml PBS and pelleted. The cell pellet was resuspended in 30 ml complete medium and seeded with a splitting ratio of 1:3. Only cells from three passages were used for the experiments. On the day of operation 2*10^6 ^cells were suspended in 100 μl complete medium for application for animals in the experimental setting A [5] (Table 1). In the experimental setting B the cell amount ranged from 1*10^4 ^to 5*10^5 ^(Table 2).

**Table 2:** Arrangement of animal groups in experiment B

Experiment B	Control/Tumor cell count	PL 9%/Tumor cell count	period
	10 rats/1*10^4^	10 rats/1*10^4^	30 days
	10 rats/5*10^4^	10 rats/5*10^4^	
	10 rats/1*10^5^	10 rats/1*10^5^	
	10 rats/2.5*10^5^	10 rats/2.5*10^5^	
	10 rats/5*10^5^	10 rats/5*10^5^	

(PL = phospholipids)

### Surgical procedure

All animals underwent a laparotomy via midline incision of 2 cm length. Before closure of the laparotomy wound tumor cells and either normal saline (controls) or phospholipid solutions with a concentration of either 6% or 9% (Fresenius, Bad Homburg, Germany) were instilled into the peritoneal cavity.

#### Experiment A

The animals in experiment A received a constant amount of 2*10^6 ^tumor cells according to our former experiment [5].

#### Experiment B

In experiment B different numbers of tumor cells (10,000, 50,000, 100,000, 250,000 and 500,000) were administered intraperitoneally with a constant amount of phospholipid emulsion (PL 9%) or normal saline in the control group.

### Phospholipids

The phospholipid solution consists of phosphatidylcholine 70% of the total weight, phosphatidylethanolamine 15% of the total weight, neutral lipids 8% of the total weight, sphingomyelin < 3% of the total weight and lysophosphatidylcholine < 3% of the total weight.

### Evaluation of peritoneal carcinosis

After intervals of 30 days, the animals were sacrificed by inhalation of a lethal dose of isoflurane. The abdomen was opened by bilateral paramedian incisions for complete exploration. The extent of peritoneal carcinosis (mm^2^) was measured using a digitizer board and calculation by costum-made software on a personal computer [12]. After subtle resection, the tumor volume (ml) was measured by water displacement. Furthermore a modified peritoneal cancer index (PCI), as described by Sugarbaker et al. [13], was determined. The original PCI was adapted concerning tumor size and areas in rats; Tumor size < 2 mm (LS-1); 2.1–5 mm (LS-2), > 5 mm or confluence (LS-3). Four areas (liver, spleen, colon, and diaphragma) were added to refine the original PCI because former animal studies showed metastases in these areas as well and evaluation confirmed more detailed information about the tumor cell dissemination. Therefore the maximum score was 51 from up to 3*17 areas. Exploration and evaluation were carried out by an independent, blinded observer [5,14].

### Statistical analysis

All data are expressed as means +/- standard error of the mean. Statistical analysis was performed by a two-way ANOVA with pairewise comparison.

## Results

### Animal experiments

Surgical treatment and injection of phospholpids showed no side effects. The mean body weight was constant. All animals developed peritoneal carcinosis during the observation period. There was no animal that was free of peritoneal implants when they were sacrificed. In group 5*10^4 ^one animal died before the end of observation period because of a viral infection.

#### Experiment A

##### 1. Intraperitoneal tumor volume

**Figure 1:**
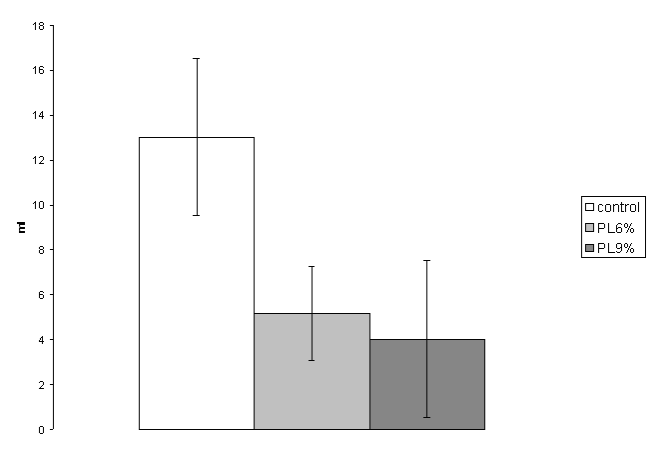
**Experiment A: Volume of intraperitoneal tumor (ml, SEM)**. (PL6% to control p = 0.001; PL9% to control p = 0.001; PL6% to PL9% no significance)

The tumor volume in the control group reached 12.0 ± 4.9 ml, in the 6% trial group 5.2 ± 2.2 ml and in the 9% trial group 4.0 ± 3.5 ml. A significant reduction of tumor volume was recognized in the 6% trial group (p = 0.001) and the 9% trial group (p = 0.001) compared to controls. The difference between 6% and 9% trial group was not significant (Figure 1).

##### 2. Area of tumor adhesion

**Figure 2:**
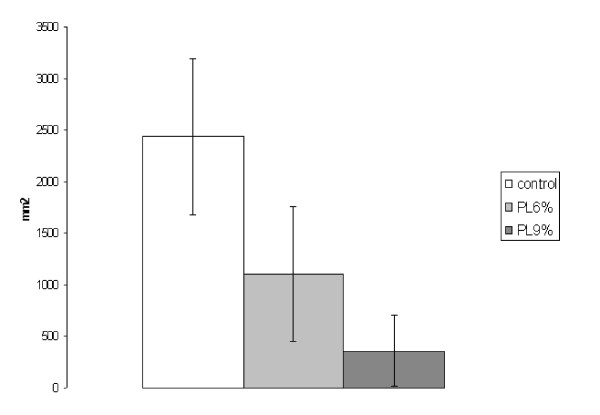
**Experiment A: Area of tumor adhesion (mm**^2^**, SEM)**. (PL6% to control p = 0.002; PL9% to control p = 0.001; PL6% to PL9% p = 0.006)

The area of tumor adhesion in the control group reached 2434.4 ± 766 mm^2^, in the 6% trial group 1106.8 ± 689 mm^2 ^and in the 9% trial group 362.7 ± 339 mm^2^. A significant reduction of the area of adhesion was assessed in both trial groups (6%: p = 0.002; 9%: p = 0.001) compared to the control group. The difference between the 6% and 9% trial group was also significant (p = 0.006) (Figure 2).

##### 3. Peritoneal cancer index

**Figure 3:**
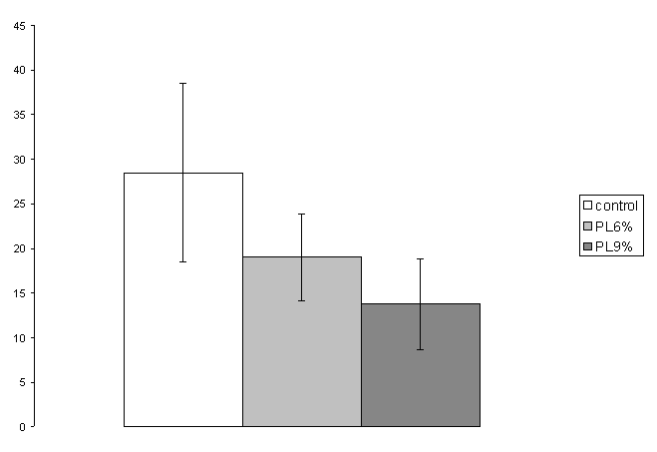
**Experiment A: Peritoneal Cancer Index (PCI, SEM)**. (PL6% to control p = 0.027; PL9% to control p = 0.001; PL6% to PL9% p = 0.048)

The PCI in the control group reached 28.5 ± 10.0, in the 6% trial group 19.0 ± 5.0 and in the 9% trial group 13.8 ± 5.1. A significant reduction of the PCI was recognized between the control group and the 6% trial group (p = 0.027) and between the control group and the 9% trial group (p = 0.001). The difference between 6% and 9% trial group was also significant (p = 0.048) (Figure 3).

#### Experiment B

##### 1. Intraperitoneal tumor volume

We found a significant reduction of tumor volume in all subgroups compared to controls. In the subgroup with the lowest tumor cell count (1*10^4^) the intraperitoneal tumor load was marginal with 0.089 ± 0.048 ml in the treated animals and 0.97 ± 0.26 ml in controls (p < 0.001).

Also in the next subgroup (5*10^4^) we found a significant reduction of the intraperitoneal tumor volume after treatment with PL 9% (1.47 ± 0.52 ml) compared to the control group (3.25 ± 1.46 ml) (p = 0.0007). After injection of 1*10^5 ^tumor cells the tumor volume could be halved in the treatment group (PL 9%: 3.27 ± 1.46 ml; control: 6.77 ± 2.47 ml) (p = 0,007).

**Figure 4:**
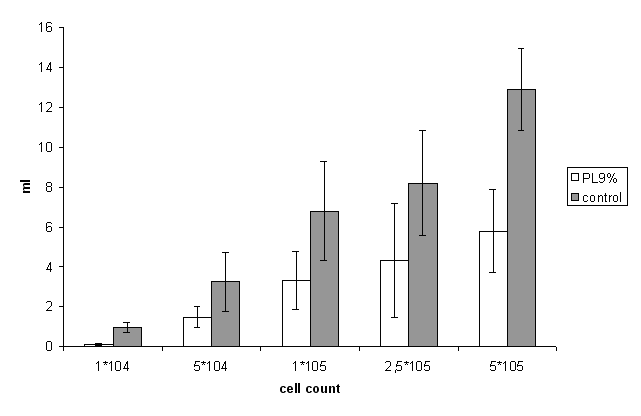
**Experiment B: Volume of intraperitoneal tumor (ml, SEM)**. (1*10^4 ^p = 0,001; 5*10^4 ^p = 0,0007; 1*10^5 ^p = 0.007; 2,5*10^5 ^p = 0,0031; 5*10^5 ^p = 0,003)

Looking to group 2.5*10^5 ^(control: 8.2 ± 2.62 ml; PL 9%: 4.34 ± 2.86 ml) we also found a significant reduction (p = 0.0031). Even in group 5*10^5^(control: 12.9 ± 2.07 ml; PL 9%: 5.75 ± 2.1 ml) the statistical analysis result in a significant reduction of tumor volume (p = 0.003) (Figure 4).

##### 2. Area of tumor adhesion

**Figure 5:**
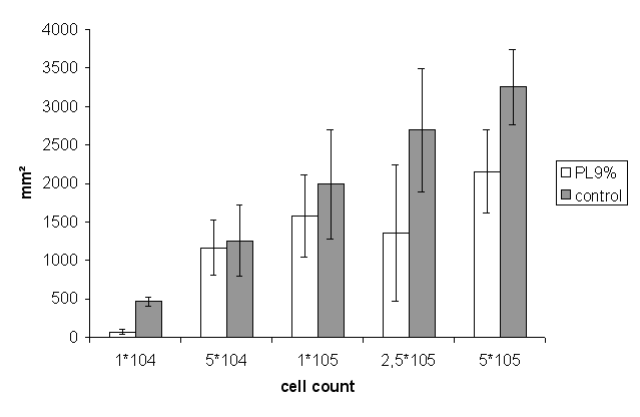
**Experiment B: Area of tumor adhesion (mm**^2^**, SEM)**. (1*10^4 ^p = 0,001; 5*10^4 ^no significance; 1*10^5 ^no significance; 2,5*10^5 ^p = 0,0032; 5*10^5 ^p = 0,0013)

Treatment with PL 9% led to a reduction of tumor adhesion in all subgroups. However, differences were only significant in the subgroups 1*10^4^(control: 464 ± 55 mm^2^; PL 9%: 65 ± 31 mm^2^) (p < 0.001), 2.5 * 10^5 ^(control: 2693 ± 801 mm^2^; PL 9%: 1354 ± 884 mm^2^) (p = 0.0032) and 5 * 10^5 ^(control: 3255 ± 489 mm^2^; PL 9%: 2151 ± 539 mm^2^) (p = 0.0013). The differences in the subgroups 5*10^4 ^(control: 1254.89 ± 460.31 mm^2^; PL 9%: 1163.54 ± 356.82 mm^2^) (p = 0.23) and 1*10^5 ^(control: 1992.07 ± 710.60 mm^2^; PL 9%: 1579.33 ± 533.02 mm^2^) (p = 0.11) did not reach statistical significance (Figure 5).

##### 3. Peritoneal Cancer Index

**Figure 6:**
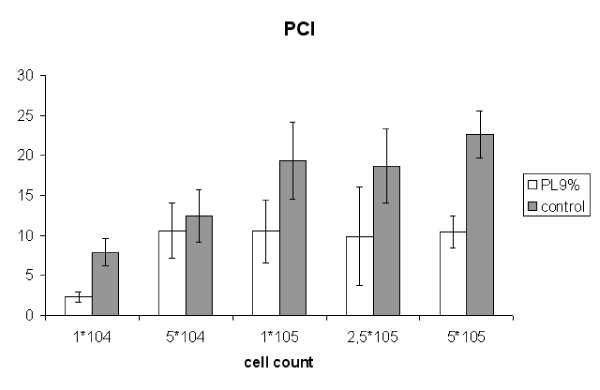
**Experiment B: Peritoneal Cancer Index (PCI, SEM)**. (1*10^4 ^p = 0,001; 5*10^4 ^no significance; 1*10^5 ^p = 0.007; 2,5*10^5 ^p = 0.001; 5*10^5 ^p = 0.0011)

The Peritoneal Cancer Index could be reduced in all consecutive subgroups. In group 1*10^4 ^(control: 7.9 ± 1.7; PL 9%: 2.3 ± 0.6) we found a significant reduction (p < 0.001). In group 5*10^4 ^(control: 12.4 ± 3.3; PL 9%: 10.6 ± 3.5) the influence of phospholipids was not significant (p = 0.17). The statistical analysis of group 1*10^5^(control: 19.3 ± 4.8; PL 9%: 10.5 ± 3.9) and 2.5*10^5^(control: 18.7 ± 4.6; PL 9%: 9.9 ± 6.2) showed a significant difference (p < 0.001 and p = 0.0011 respectively). In group 5*10^5 ^treatment with PL 9% also resulted in a significant reduction of the PCI with a value of 10.4 ± 2.0 compared to the control group (22.6 ± 2.9) (p < 0.001) (Figure 6).

## Discussion

The invasion of malignant tumor cells is determined by the capability to migrate, adhere to and degrade ECM components [15]. This study was focused on the adhesion of tumor cells and on the preventive influence of phospholipids.

We used an established experimental model of peritoneal carcinosis after intraabdominal instillation of tumor cells (Cell line: DHD/K12/TRb) into female BD-IX rats [5,16]. All animals in this experiment developed peritoneal metastases. The effects could reliably be described by the area of attachment, the tumor volume and the PCI.

The idea of inhibiting intraperitoneal tumor nidation is not new. Several intraperitoneal therapies have been tested. Hagiwara et al. examined the anti-adherent effect of dextran sulphate after tumor implantation in mice [17]. A prolonged survival in the treatment group after inoculation of melanoma cells was described. Moreover, intraabdominal tumor growth of CC531 adenocarcinoma cells in rats undergoing laparoscopy could be diminished using low-molecular-weight heparin in combination of intraperitoneal lavage und subcutaneous injection [18]. Ogaswara et al. [19] have described the inhibition of tumor invasion and growth testing intraperitoneal applied antioxidants like epigallocatechin gallate (EGCG) in a colon 26-L5 adenocarcinoma mice model. Treating carcinomatosis of colorectal cancer by cytoreduction and hyperthermic intraperitoneal chemotherapy have been studied by Zoetmulder et al. [20]. They calculated the survival after cytoreduction and perfusing the abdomen with mitomycin C (35 mg/m^2^) at 40 degrees C to 41 degrees C for 90 minutes. They observed a survival benefit for colorectal cancer patients with peritoneal carcinomatosis [21]. Regarding angiogenesis as another key step in tumor growth, invasion and metastasis, antiangiogenic is an additional approach for antitumor treatment. Thus Nestler et al. [22] have investigated the effect of angiostatin on the growth of CC531 colon carcinoma cells in vitro and in a laparoscopic animal model of peritoneal carcinomatosis. They found a significantly diminished intraperitoneal tumor growth in rats after intraperitoneal application of 20 mg angiostatin.

Tumor cells are known to stimulate peritoneal fibrosis, creating a congenial environment for peritoneal metastasis [10,23]. Phospholipids are capable of forming a remarkably resistant lubricant layer on the peritoneal surface [24-27]. We suggest that the ability of phospholipids to cover peritoneal defects with exposed extracellular matrix subsequently inhibits tumor cell attachment [5]. Intraperitoneal phospholipids have already been used to prevent postoperative adhesion. They showed a significant reduction of adhesion formation [10,25,26]. The authors found no adverse side effects and no impairment of healing of anastomosis, laparotomy wounds and liver incisions after intraabdominal treatment with phospholipids [10,26]. We examined the effect of intraperitoneal phospholipids in view of the tumor cell adhesion [5]. In former studies we could reliable demonstrate a significant reduction of tumor cell adhesion in case of a constant tumor cell concentration (1*10^6 ^tumor cells) [5,14]. With respect to the idea using phospholipids as an adjuvant intraperitoneal therapy in case of standard operations we wanted to examine the effect in case of lower tumor cell concentrations.

In *experiment A *we could demonstrate a significant reduction of peritoneal dissemination as measured by all evaluation methods after treatment with 6%- and 9%-phospholipids. In former studies -using the same tumor cell concentrations but lower phospholipid concentrations (1,5% und 3%)- we found a reduction of peritoneal dissemination, too. However, the difference between control-group and treatment-group was not as distinctive as in this case [5]. In *experiment B *we diversified the number of tumor cells to mimic the clinical situation, that during the resection of a colon cancer only very few tumor cells were released.

There was a remarkable reduction of peritoneal dissemination especially in low tumor cell concentrations, supporting our theory of treating every patient with a gastrointestinal tumor at the end of the operation with phospholipids to avoid adhesions and peritoneal tumor dissemination. The absence of statistical significance in group 5*10^4 ^evaluating PCI and "area of tumor adhesion" possibly can be traced back to a low falling number because of the dead of an animal in this group. Valuating the results of the underlaying study we have to stress that the extent and not the incidence of peritoneal carcinomatosis was reduced. One also has to be aware that our results derived from a rat model attending only one kind of gastrointestinal cancer.

This in vivo model of intraperitoneal application of phospholipids to decrease the tumor cell adhesion describes the effect from a macroscopic and clinical point of view. Former studies could exclude a cytotoxic effect of the used phospholipids [25,28,29]. However, various studies could demonstrate alterations in the adhesive properties of tumor cells depending on the degree of differentiation indicating a change of integrin expression [30-33]. According to this further studies are currently in progress to evaluate the effect of phospholipids on the structure of cell membranes.

## Conclusion

In this experimental animal study, intraperitoneal treatment with phospholipids resulted in reduction of the extent of peritoneal carcinomatosis after intraperitoneal administration of free tumor cells. This effect was exceptionally noticed when the amount of intraperitoneal tumor cells was limited. Consequently, intraperitoneal administration of phospholipids might be effective in reducing the incidence and extent of peritoneal carcinomatosis after surgery of gastrointestinal tumors in humans.

## Competing interests

The auhor(s) declare that they have no competing interests.

## Pre-publication history

The pre-publication history for this paper can be accessed here:


